# Data on genome sequencing, assembly, annotation and genomic analysis of *Rhodococcus rhodochrous* strain SPC17 isolated from Lonar Lake

**DOI:** 10.1016/j.dib.2020.105336

**Published:** 2020-02-26

**Authors:** Satish Kumar, Dhiraj Paul, Yogesh Shouche, Mangesh Suryavanshi

**Affiliations:** a National Centre for Cell Science, Savitribai Phule Pune University Campus, Pune, MH, India; b ICAR-National Institute of Abiotic Stress Management, Baramati, Pune, MH, India; c Yenepoya Research Centre, Yenepoya Deemed to be University, Mangalore, 575018, Karnataka, India

**Keywords:** *Rhodococcus*, Soda lake, Draft genome sequence, Biodegradation, Hydrocarbon

## Abstract

The bacterial isolates of genus *Rhodococcus* are best known for their significant biodegradation abilities. Here, we report the data related to draft genome sequencing of *Rhodococcus rhodochrous* strain SPC17 isolated from sediments of Lonar Lake. The *de novo* assembly of 1598096 Illumina's paired-end sequencing reads resulted in 51 contigs for an overall genome assembly size of 4.98Mb. A total of 4546 genes were predicted using the National Center for Biotechnology Information- Prokaryotic Genome Annotation Pipeline (NCBI-PGAP). RAST server-based annotation of the *Rhodococcus* strain SPC17 genome resulted in a total of 295 subsystems with 25% subsystem coverage. The data on the draft genome shotgun project are accessible at NCBI-GenBank under the accession number WUUR00000000. Our data resource will facilitate further molecular and genomic studies of diverse hydrocarbon catabolizing genes present in *Rhodococcus rhodochrous* strain SPC17.

**Table:** 

Subject	Biology
Specific subject area	Genomics
Type of data	Raw Illumina's NGS paired-end sequencing reads in FASTQ format. Genome assembly data and assembled contigs in FASTA format. Data related to predicted genes and annotation of respective proteins.
How data were acquired	Shotgun genome sequencing using Illumina Novaseq platform (2 × 150 paired-end chemistry) followed by *de novo* genome assembly and annotation.
Data format	Raw data FASTQ format (NGS raw reads) and Data on assembled contigs and predicted genes (FASTA format).
Parameters for data collection	Genomic DNA was extracted from a pure culture of *Rhodococcus rhodochrous* SPC17.
Description of data collection	Whole-genome sequencing, genome assembly, and annotation
Data source location	*Rhodococcus rhodochrous* SPC17 was isolated from sediment samples of Lonar Lake, located in Aurangabad district, Maharashtra, India. (19°58′34″ N and 76°30′30″ E).
Data accessibility	The draft genome sequence of *Rhodococcus rhodochrous* SPC17 has been deposited at NCBI- GenBank under the accession WUUR00000000. Whole-genome can be accessed using the given below link (https://www.ncbi.nlm.nih.gov/nuccore/WUUR00000000). “NCBI-GenBank: WUUR00000000” Raw NGS sequence data are also deposited in NCBI-SRA (Short Read Archive) with accession number ‘SRX7445443’ and can be accessed using the following link (https://www.ncbi.nlm.nih.gov/sra/SRX7445443[accn]). All the details about the genome sequencing project are available on NCBI under BioProject accession number PRJNA597860 and can be accessed using the following link (https://www.ncbi.nlm.nih.gov/bioproject/PRJNA597860)

**Table:** 

**Value of the Data** • The draft genome sequence data of *Rhodococcus rhodochrous* strain SPC17 can be used to design PCR primers to study the diverse catabolic genes and also to design the genetic engineering-based strategies for reducing damages caused by hydrocarbon contamination. • The genome sequence data will be useful for the comparative genomic studies for exploring the core and the pangenome of the genus *Rhodococcus.* • Whole genome sequence data will be useful for studying the genomic basis of the survival of this bacterium in high salt and alkaline pH conditions of Soda Lake.

## 1 Data description

Bacterial isolates belonging to genus *Rhodococcus* are best known for their remarkable catabolic versatility and their ability to degrade a wide variety of aliphatic, aromatic, and polyaromatic hydrocarbons [[1], [2], [3]]. Taxonomically genus *Rhodococcus* belongs to the *Nocardiaceae* family under phylum Actinobacteria. The immense catabolic diversity and remarkable biotransformation capabilities of *Rhodococcus* have been largely attributed to the presence of a diverse array of catabolic genes encoding for several enzymatic classes like monooxygenases, dioxygenases, and hydroxylases [4]. In this data article, we present a description of genome sequencing data and the draft genome of *Rhodococcus rhodochrous* strain SPC17 originally isolated from sediments of Lonar Lake located in the western plateau region of Maharashtra province of India. This soda lake is well-known for its high saline and hyper-alkaline conditions and represents the only existing meteorite impact crater in the world created in the basaltic rocks [5].

**Fig. 1:**
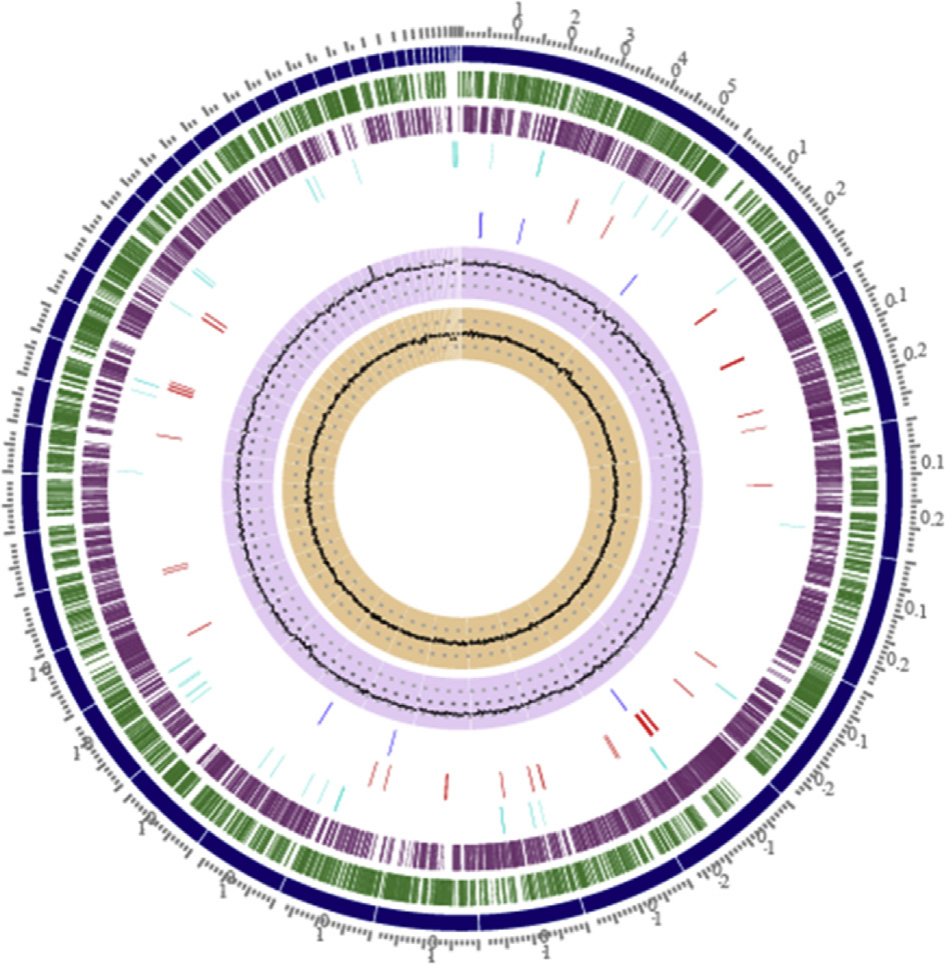
Graphical circular view of the genome map of *Rhodococcus rhodochrous* strain SPC17. The tracks in the figure are displayed as concentric rings, from outermost to innermost tracks representing the 1-Reference position in the genome, 2-Position and order of the 51 assembled contigs, 3- CDS- forward strands, 4-CDS- reverse strand, 5-Non-coding features, 6-AMR genes (Anti-microbial Resistance), 7-Genes for Transporters, 8- GC content and 9- GC skew respectively.

The DNA was extracted from the pure culture of *R*. *rhodochrous* strain SPC17 and the genome sequencing was performed using the Illumina NovaSeq 6000 platform. A total of 1598096 paired-end sequencing reads were generated using Illumina's 2 × 150 bp sequencing chemistry. The *de novo* genome assembly of 1598096 Illumina's paired-end sequencing reads resulted in 51 contigs for an overall genome assembly size of 4.98Mb (4,985,784 bases). The quality parameters for the genome assembly such as N50 value (197398 bases), coarse consistency (97.6%), and fine consistency (96.1%) indicated the good quality of the assembled genome as evaluated using CheckM [6]. The genome of the *Rhodococcus* strain SPC17 has a high GC content of 68.39%. Using NCBI-PGAP (Prokaryotic Genome Annotation Pipeline) for genome annotation, it was determined that the genome contains 4484 coding sequences (CDSs), 4 rRNAs genes (1 copy of 5S, 1 copy16S, and 2 copy of 23S, respectively), 55 tRNAs, 3 ncRNAs (non-coding RNAs), and 60 pseudogenes. The genomic features of *Rhodococcus* strain SPC17 are represented in the circular map of the genome as shown in Fig. 1.

**Fig. 2:**
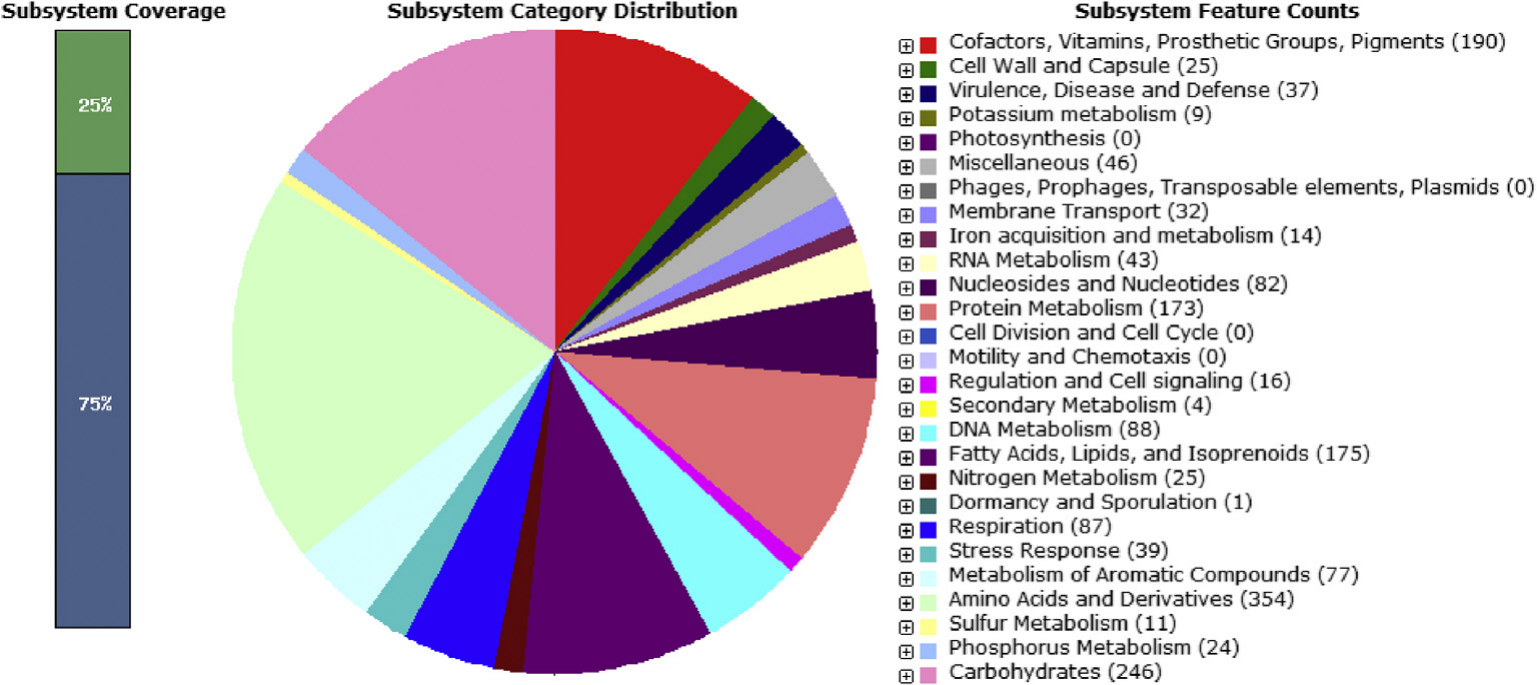
An overview of the subsystem categories assigned to the genes predicted in the genome of *Rhodococcus rhodochrous* SPC17. The whole-genome sequence of the strain SPC17 was annotated using the RAST server.

The RAST server-based annotation of the *Rhodococcus* strain SPC17 genome resulted in a total of 295 subsystems with 25% subsystem coverage (Fig. 2). The subsystem category distribution of the genes assigned to different subsystems indicated the highest genes assigned to metabolism of amino acids and derivatives (354 genes), followed by the metabolism of carbohydrates (246 genes), cofactors, vitamins, prosthetic group, and pigments (190 genes), fatty acids, lipids, and isoprenoids (175 genes) and protein metabolism (173 genes). RAST based annotation classified 77 genes in subsystem category ‘metabolism of aromatic compounds’ and 39 genes in subsystem category ‘stress response’. The 77 genes assigned to subsystem category ‘metabolism of aromatic compounds’ included genes related to Quinate degradation (2 genes), Biphenyl Degradation (10 genes), Benzoate degradation (6 genes), and p-Hydroxybenzoate degradation (1 gene), Catechol branch of beta-ketoadipate pathway (12 genes), Salicylate and gentisate catabolism (6 genes), Protocatechuate branch of beta-ketoadipate pathway (14 genes), Homogentisate pathway of aromatic compound degradation (21 genes), and Gentisate degradation (5 genes). The 12 genes assigned to branched ketoadipate pathway in *Rhodococcus* strain SPC17 implies its ability to catabolize the benzoate and phthalate compouds. Total 39 genes were assigned to subsystem category ‘Stress response’ and the analysis of the genes for this subsystem category revealed the presence of genes *betA* (encoding for *Choline dehydrogenase* (EC 1.1.99.1), *betT* (encoding a high-affinity choline uptake protein BetT) and *opuD* (encoding for glycine betaine transporter OpuD). This implies the ability of the isolate to synthesize and uptake ‘Glycine betaine (N,N,N-trimethylglycine)’ which may be the main osmolyte synthesized by this bacterium under osmotic stress conditions usually present in the soda lake environment. The details of the RAST-based annotation are given in supplementary sheet-S1. The genomic data reported here will pave the way for further study of the different catabolic genes implicated in hydrocarbon metabolism in this bacterium.

## 2 Experimental design, materials, and methods

### 2.1 Bacterial strain isolation and identification

The bacterial isolate SPC17 was isolated from the sediment sample of Lonar Lake on Standard Plate Count (SPC) microbiological medium as described by Watve and coworkers [7]. The strain SPC17 was identified as *Rhodococcus rhodochrous* based on the mass spectra of the cell extracts of overnight grown pure cultures following the method described by Pandey and coworkers [8]. The mass spectra were acquired in a linear positive ion extraction mode within a mass range from 2000 to 20,000 Da. using AUTOFLEX TOF/TOF Mass spectrometer (Bruker Daltonik GmbH, Germany). The MALDI Biotyper software 3.0 (Bruker Daltonik) was used to visualize and match the mass spectra which resulted in the identity of the pure culture as *Rhodococcus rhodochrous* with the biotyper score value 2.2.

### 2.2 Genome sequencing, assembly, and annotation

For genome sequencing, bacterial DNA was extracted from an overnight grown pure culture of *Rhodococcus rhodochrous* strain SPC17 using MoBio microbial DNA extraction kit (MoBio Laboratories). The DNA quality was checked on 1.0% agarose gel. The library preparation for Illumina sequencing was performed using the TruSeq DNA Library Preparation Kit (IlluminaInc., USA) according to the manufacturers instructions. The library insert sizes selected were 300 bases for Illumina sequencing. The genome was sequenced using the Illumina's Novaseq platform with 2 × 150 paired-end chemistry. The quality of the raw reads was analyzed by FastQC software [9]. Trimming of the raw reads was done by Trimmomatic 0.32 software [10] and only the good quality reads were assembled using *de novo* assembler SPAdes 3.10.0 [11]. The annotation was carried out with the National Center for Biotechnology Information- Prokaryotic Genome Annotation Pipeline (NCBI PGAP) [12] through the NCBI Genome submission portal (Genome Submit at http://ncbi.nlm.nih.gov). A genome circular map was created using the ‘circular viewer’ functionality implemented in the PATRIC web server [13]. The mapping of genes to subsystems and metabolic reconstruction of the high-quality genome annotations were obtained using Rapid Annotation using the Subsystem Technology (http://rast.nmpdr.org) [14].

## Author contributions

Conceived and designed the experiments: DP, YS, SK. Wet lab experiment: SK, DP, MVS, Data analysis, and interpretation: SK, DP, Manuscript preparation: SK, DP. Contributed reagents/materials/analysis tools: YS, MVS. All authors read and approved the final manuscript.
